# Nonvolatile Bio-Memristor Based on Silkworm Hemolymph Proteins

**DOI:** 10.1038/s41598-017-17748-6

**Published:** 2017-12-12

**Authors:** Lu Wang, Dianzhong Wen

**Affiliations:** HLJ Province Key Laboratory of Senior-education for Electronic Engineering, Heilongjiang University, Harbin, Heilongjiang 150080 China

## Abstract

This paper reports the first successful fabrication of an ITO/silkworm hemolymph/Al bio-memristor using silkworm hemolymph as the active layer. Experiments demonstrate that the silkworm hemolymph bio-memristor is a nonvolatile rewritable bipolar memory device with a current switching ratio exceeding 10^3^. The state of the bio-memristor can be retained for more than 10^4^ seconds and remains stable for at least 500 cycles. Tests of 1/f noise have shown that the resistance switching characteristics of the silkworm hemolymph bio-memristor are related to the formation and breaking of conductive filaments, which result from the migration of oxygen ions and the oxidation and reduction of metal cations in the silkworm hemolymph film. The naturally non-toxic silkworm hemolymph offers advantages for human health, environmental protection, and biocompatibility. The proposed nonvolatile rewritable bio-memristor based on silkworm hemolymph possesses great application potential.

## Introduction

Because memristors have extensive and important application prospects in many areas, including information storage, logical calculation, and artificial neural networks, they have recently become a research hotspot in many fields, such as materials, physics, electronics and biology^1–3^. Various types of materials have been reported for the production of memristors, including biological materials (e.g., egg white^4,5^ and ferritin^6^), binary oxide materials (e.g., TaO_x_^7^, HfO_x_^8^ and FeO_x_^9^), organic and polymeric materials^10,11^, carbon-based materials (e.g., graphene^12^ and carbon nanotubes^13^) and silicon-based materials^14,15^.

Relative to other materials, biological memristors fabricated from natural non-toxic biological materials have advantages related to human health, environmental protection, biocompatibility, and convenience of manufacturing and are low cost. Thus, they have been extensively studied during the past few years. For example, a current switching ratio of 10^3^ was reported for a bio-memristor fabricated from egg white^4^. The memory mode and threshold of a Pt/ferritin/Pt memristor fabricated from ferritin was adjusted by modulating the limiting current^6^. Furthermore, a Au/starch/ITO memristor was reported to have a low operating voltage. When the active layer is a composite of starch and chitosan, the resistance switching characteristic gradually changes^16^. The silk fibroin solution extracted by degumming and purifying cocoons has been used to fabricate memristors with relatively low ON/OFF current ratios of approximately 10 at 4 V^17^. The incorporation of Au nanoparticles was found to significantly improve the current switching ratio of a fibroin-based memristor^18^. The resulting memristor, which was fabricated from the sericin extracted from cocoons and Au nanoparticles, exhibited multi-memory modes^19^. Additionally, memory devices made from spider silk have been reported to have current switching ratios of approximately 60^20^.

In this work, silkworm hemolymph is identified as a biomaterial that can be used to fabricate memristors. As a natural, non-toxic biological material, silkworm hemolymph is compatible with human health and environmental protection. We successfully fabricated ITO/silkworm hemolymph/Al bio-memristors by creating the active layer from silkworm hemolymph. The experimental results show that the silkworm hemolymph bio-memristor has a bistable resistance switching characteristic with a high ON/OFF current ratio above 10^3^ and exhibits rewritable flash memory. Additionally, the silkworm hemolymph bio-memristor can endure over 500 write-read-erase-read cycles, and its retention time can exceed 10^4^ s. The material used for the active layer of this bio-memristor is different from that used for the memristors investigated in our previous studies^21–23^.

## Results and Discussion

**Figure 1:**
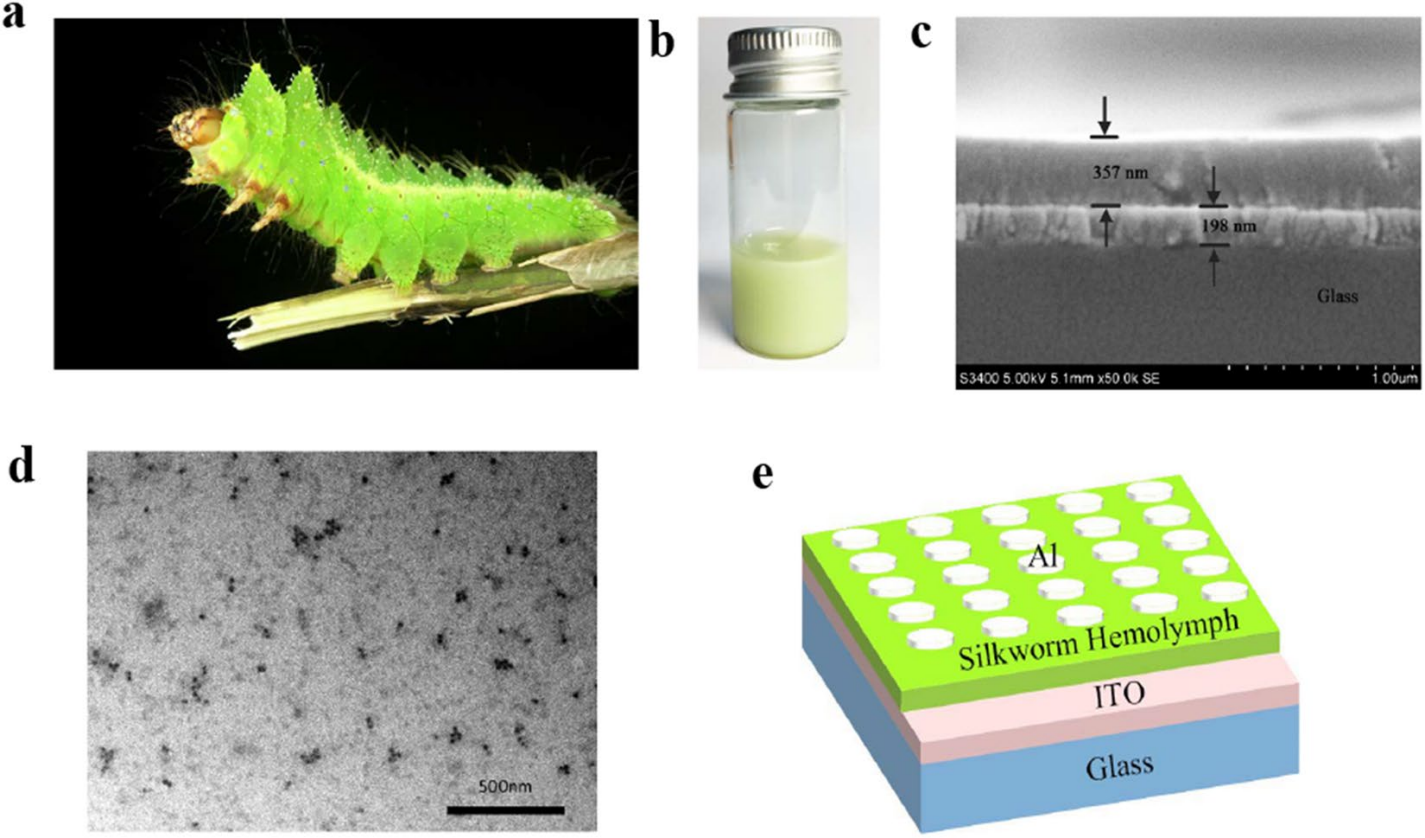
(**a**) A photograph of a silkworm (*Antheraea pernyi*) larva. (**b**) Silkworm hemolymph obtained directly from silkworm larvae. (**c**) Cross-sectional SEM image of a silkworm hemolymph film and an ITO film on a glass substrate. (**d**) TEM image of silkworm hemolymph. (**e**) Schematic illustration of the silkworm hemolymph bio-memristor.

The silkworm is a metamorphic insect, and its life consists of four growth stages: egg, larva, pupa and adult (i.e., moth). The silkworm morphology and physiological function are completely different in each stage of silkworm development. More than seven hundred types of proteins have been identified in various stages of the silkworm life cycle, most of which are 30 K proteins and SP-1 and SP-2 storage proteins^24–27^. The silkworm circulatory system consists of open blood circulation, and its blood cavity comprises the entire body cavity. The silkworm hemolymph consists of two components: blood and lymph^24^. The silkworm hemolymph used in this work was obtained directly from silkworm (*Antheraea pernyi*) larvae. Figure 1a and b show photographs of a silkworm (*Antheraea pernyi*) larva and the silkworm hemolymph, respectively. The silkworm hemolymph was spin-coated onto ITO glass, and the glass was then dried in a drying oven and characterized by a scanning electron microscope (SEM), as shown in Fig. 1c. The order of the layers in the SEM images from top to bottom is as follows: silkworm hemolymph film, ITO film and glass. The thickness of the silkworm hemolymph film is 357 nm, and the thickness of the ITO film is 198 nm. Transmission electron microscopy was used to observe the micro-structural properties of the silkworm hemolymph, as shown in Fig. 1d. Finally, an aluminum electrode with a thickness of 180 nm was deposited on the silkworm hemolymph film at a pressure of 1.2 × 10^−4^ Pa by thermal evaporation. The diameter of a single aluminum electrode is 300 μm. The aluminum electrode is the upper electrode of the silkworm hemolymph bio-memristor, and the bare ITO electrode is the lower electrode. A schematic illustration of the silkworm hemolymph bio-memristors is shown in Fig. 1e.

**Figure 2:**
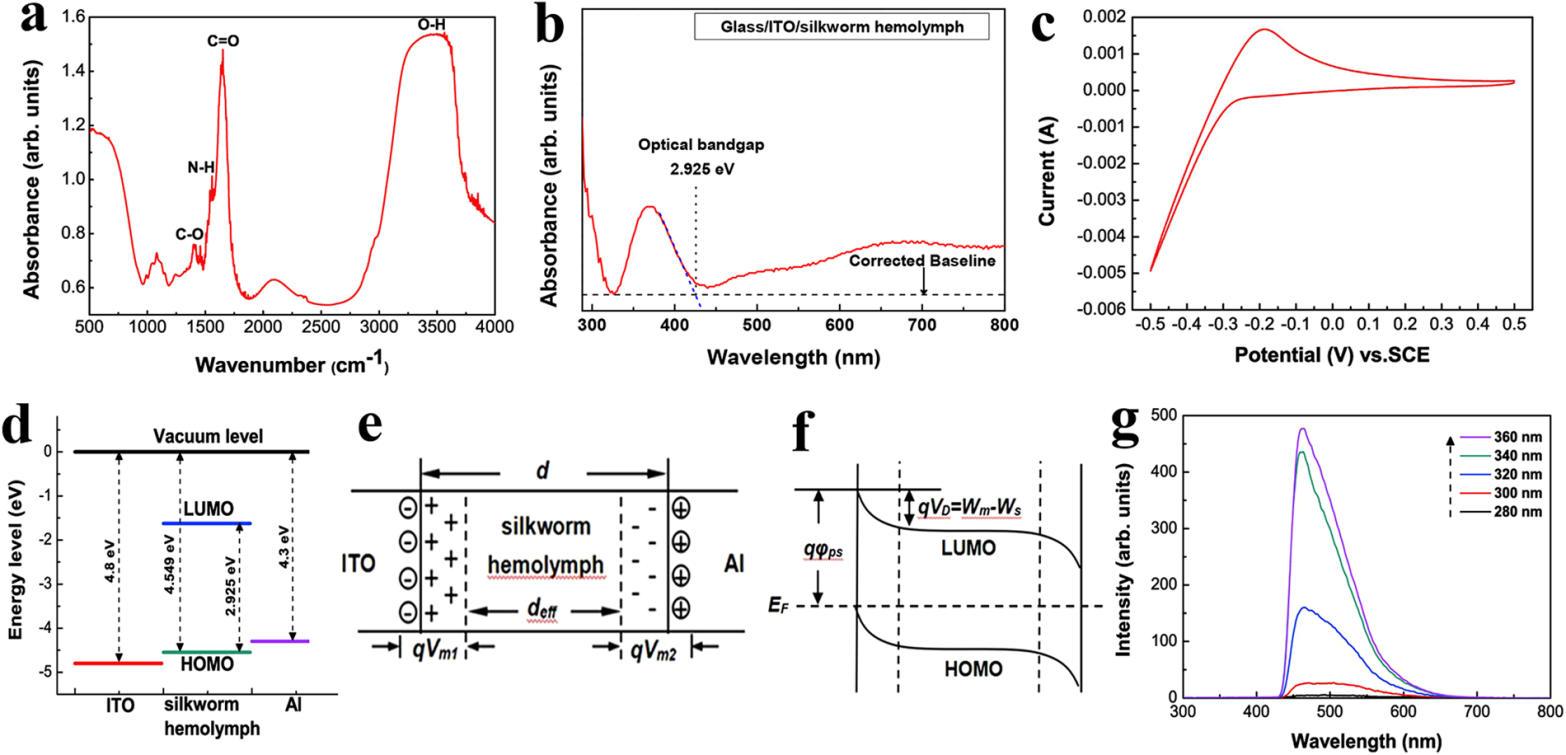
(**a**) Infrared spectrum of the silkworm hemolymph. (**b**) UV-vis absorption spectrum of the silkworm hemolymph film used to calculate the band gap width. (**c**) Cyclic voltammogram of the silkworm hemolymph film. (**d**) Diagram of the energy levels of the materials used in the ITO/silkworm hemolymph/Al bio-memristor. (**e**) One-dimensional structure model and charge distribution diagram for the ITO/silkworm hemolymph/Al bio-memristor. (**f**) Energy band diagram of the ITO/silkworm hemolymph/Al bio-memristor. (**g**) Fluorescence emission spectra of the silkworm hemolymph film excited at different excitation wavelengths.

Infrared spectroscopy was performed to analyze the chemical bonds in the silkworm hemolymph. Figure 2a presents the infrared spectrum of the silkworm hemolymph. A small absorption peak appears at 1399 cm^−1^, which is related to the carboxylate (C-O) moiety. The absorption peak at 1558 cm^−1^ is attributed to the amide (N-H)^28,29^. A relatively sharp peak appears at 1652 cm^−1^ and corresponds to the carboxyl (C=O) group^30^. A relatively wide peak related to the hydroxyl (OH, ~ 3500 cm^−1^) group spans from 2600 cm^−1^ to 4000 cm^−1^ in the spectrum^28,31^. To calculate the band gap width E_g_ of the silkworm hemolymph, the UV-vis absorption spectrum of the silkworm hemolymph film was measured, as shown in Fig. 2b. The wavelength corresponding to the band gap width of the silkworm hemolymph can be calculated from the intersection of the absorption onset line and the corrected baseline^32^. At this point, λ is calculated to be 424 nm. According to E_g_ = hc/λ, the band gap width of the silkworm hemolymph was computed to be 2.925 eV.

The electrochemical analysis of the silkworm hemolymph film is presented in Fig. 2c and d. Figure 2c shows the cyclic voltammogram of the silkworm hemolymph film in hydrochloric acid solution (0.02 mol/L). The onset oxidation potential *Eox* vs. the saturated calomel electrode is −0.191 V. The highest occupied molecular orbital (HOMO) and lowest unoccupied molecular orbital (LUMO) of the silkworm hemolymph are expressed as follows^33^:

1 $${E}_{HOMO}=-4.74-{E}_{ox}$$

2 $${E}_{LUMO}={E}_{HOMO}+{E}_{g}$$

*E*_*HOMO*_ (−4.549 eV) and *E*_*LUMO*_ (−1.624 eV) of the silkworm hemolymph were determined. The diagram of the energy levels of the materials used in the ITO/silkworm hemolymph/Al bio-memristor is shown in Fig. 2d. A one-dimensional structure model and a charge distribution diagram for the ITO/silkworm hemolymph/Al bio-memristor are shown in Fig. 2e, and the energy band diagram of the ITO/silkworm hemolymph/Al bio-memristor is shown in Fig. 2f. Here, *d* is the thickness of the silkworm hemolymph film; *d*_*eff*_ is the thickness of the effective layer, i.e., the thickness of the undoped region; *V*_*m1*_ and *V*_*m2*_ are the voltage drops in the doped region on the ITO electrode side and on the Al electrode side, respectively; *qφ*_*ps*_ is the barrier height on the side of the metal electrode; *qV*_*D*_ is the barrier height on the side of the silkworm hemolymph film; *W*_*m*_ and *W*_*s*_ are the work functions of the metal and the silkworm hemolymph, respectively; and *E*_*F*_ is the Fermi level.

To further analyze the optical characterization of the silkworm hemolymph film, a detailed photoluminescence study with different excitation wavelengths was conducted. The measured fluorescence emission spectra of the silkworm hemolymph film obtained with excitation wavelengths of 280, 300, 320, 340, and 360 nm are shown in Fig. 2g. The test results reveal that the fluorescence spectra of the silkworm hemolymph film are dependent on the excitation wavelength. The fluorescence intensity gradually increases as the excitation wavelength increases from 280 to 360 nm.

**Figure 3:**
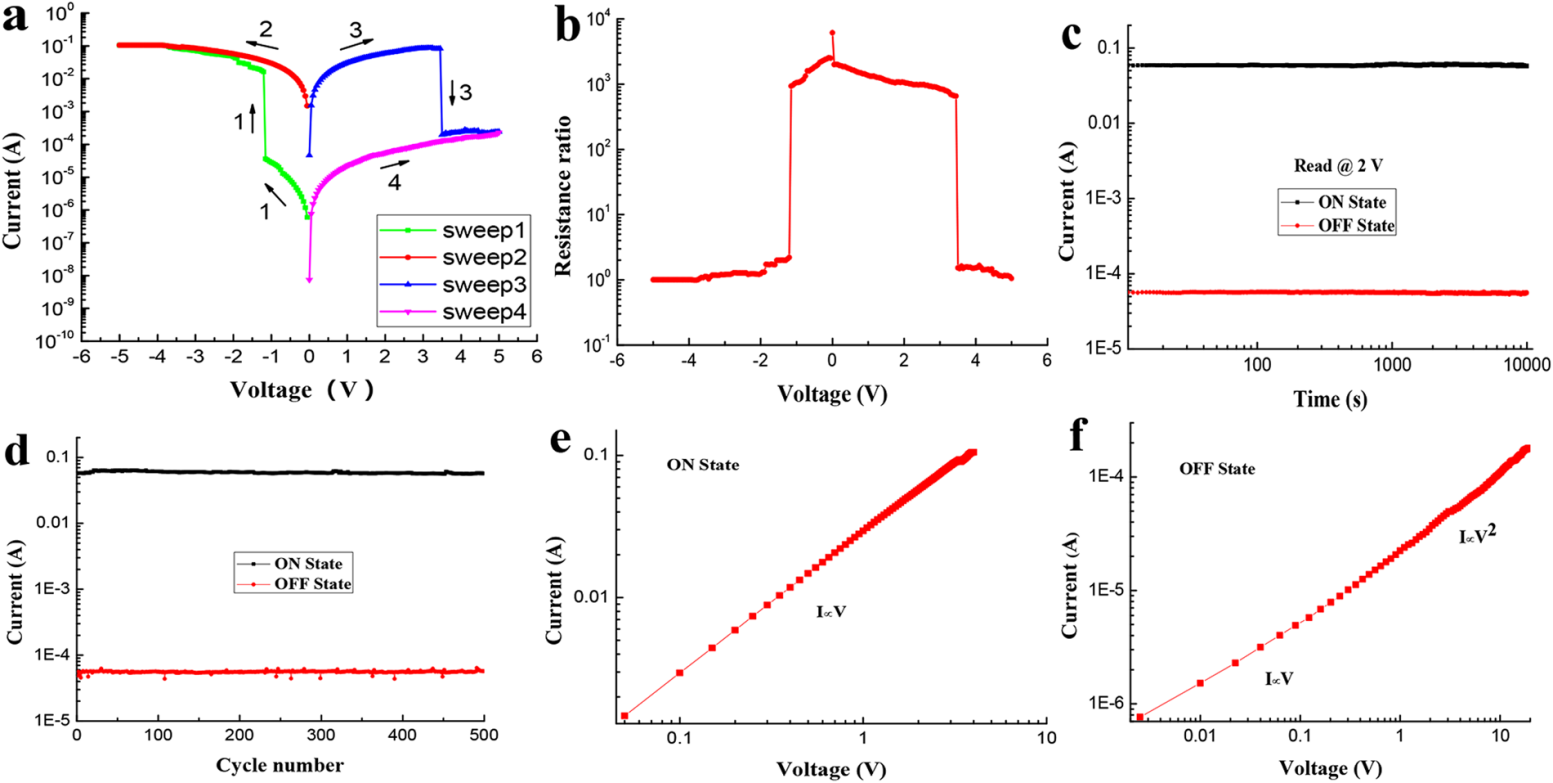
(**a**) I-V characteristics of the silkworm hemolymph bio-memristor. (**b**) The variation of the ON/OFF resistance ratio of the silkworm hemolymph bio-memristor as the voltage changes. (**c**) Retention and (**d**) endurance performances of the silkworm hemolymph bio-memristor. (**e**)–(**f**) I-V characteristics of the silkworm hemolymph bio-memristor in the (**e**) low- and (**f**) high-resistance states in double logarithmic coordinates.

The current-voltage characteristics of the silkworm hemolymph bio-memristor were measured using a semiconductor characterization system. For these measurements, the ITO electrode was grounded, and a voltage was applied to the Al electrode. The scan step was set to 0.05 V, and the limit current was set to 0.1 A. Figure 3a shows the current-voltage characteristics of the silkworm hemolymph bio-memristor. The arrows in the figure indicate the order and direction of the voltage sweeps. During the first sweep (from 0 to −5 V), the current suddenly increased from 10^−5^ to 10^−2^ A at a threshold voltage of −1.2 V. Thus, the bio-memristor was converted from the OFF state to the ON state (i.e., the writing operation of the bio-memristor). During the second sweep (from 0 to −5 V), the bio-memristor remained in the ON state. During the third sweep (from 0 to 5 V), the current suddenly dropped at 3.5 V, and the bio-memristor was converted from the ON state to the OFF state (i.e., the erase operation of the bio-memristor). During the fourth sweep (from 0 to 5 V), the bio-memristor remained in the OFF state. These sweeps constitute a complete write-read-erase-read cycle of the bio-memristor. The experimental results show that the silkworm hemolymph bio-memristor has a bistable resistance switching characteristic with an ON/OFF current ratio exceeding 10^3^ at 2 V and exhibits rewritable flash memory.

Figure 3b shows the variation of the ON/OFF resistance ratio as the voltage changes for the silkworm hemolymph bio-memristor. An ON/OFF resistance ratio exceeding 2 × 10^3^ at 0.1 V was achieved. The high ON/OFF resistance ratio of the bio-memristor indicates that this device has great potential applicability in the field of resistive random access memory. The retention performance and endurance performance of the silkworm hemolymph bio-memristor were measured. Figure 3c shows the currents of the bio-memristor in the ON state and OFF state at 2 V. During a long test (10^4^ s), the ON/OFF current ratio of the silkworm hemolymph bio-memristor remained higher than 10^3^ at 2 V, and the current did not decrease significantly. Figure 3d presents the endurance performance of the silkworm hemolymph bio-memristor. The writing operation and erasing operation were completed by applying pulse signals of −2 V/100 ms and 4 V/100 ms, respectively. The current of the bio-memristor after every operation was measured at 2 V. The test results indicated that the bio-memristor exhibited favorable rewritable performance over 500 switching cycles.

Table 1 compares the characteristics of different memristors fabricated from silkworm hemolymph (this study) and various other biological materials. As shown in Table 1, both the ON/OFF current ratio and the retention and endurance performances of our silkworm hemolymph bio-memristor are superior to those of the other memristors fabricated from biological materials.

**Table 1:** Comparison of the characteristics of different memristors: silkworm hemolymph and other biological materials.

Device structure	I_on/off_ ratio	Endurance [cycles]	Retention [s]	Ref.
ITO/Silkworm Hemolymph/Al	>10^3^	>500	>10^4^	This work
Al/Egg White/ITO	>10^3^	~500	>10^4^	4
Al/Silk Fibroin (degummed from cocoons)/ITO	~10	~120	>900	17
Ag/Fibroin (from spider silk)/Au/Si	~60	~100	>10^3^	20
Ag/Pectin/FTO	~450	~100	>10^3^	38
Au/DNA/Au	~30	~100	~10^5^	39

To analyze the conductive model of the silkworm hemolymph bio-memristor, the I-V characteristics of the device were redrawn in double logarithmic coordinates (Fig. 3e and f). Figure 3e shows the I-V characteristics of the bio-memristor in the low-resistance state. The slope of the log I-log V characteristic curve is 1, reflecting ohmic conduction behavior. The equation describing ohmic conduction is

3 $$J\propto Vexp(\frac{-{\rm{\Delta }}{E}_{ae}}{kT})$$

where *V* is the electric field, ∆*E*_*ae*_ is the electron activation energy, *k* is Boltzmann’s constant, and *T* is the temperature. This observation indicates that the relationship between the current and voltage follows Ohm’s law when the bio-memristor is in a low-resistance state. Figure 3f shows the I-V characteristic of the bio-memristor in a high-resistance state. The slope of the log I-log V characteristic curve is 1 in the low-voltage region, reflecting ohmic conduction behavior. In contrast, the slope of the curve is approximately 2 in the high-voltage region, indicating that the relationship between the current and the voltage gradually follows Child’s law; that is, the current can be fitted by a space-charge-limited current (SCLC).

4 $$J\propto \frac{9\mu {\varepsilon }_{r}{\varepsilon }_{0}{V}^{2}}{8{d}^{3}}$$

where *μ* is the mobility of the charge carriers, *ε*_*r*_ is the relative dielectric constant of the silkworm hemolymph, *ε*_0_ is the permittivity of free space, and *d* is the thickness of the silkworm hemolymph film. Thus, the conductive models of the silkworm hemolymph bio-memristor in low- and high-resistance states differ. In the low-resistance state, the higher conductivity of the bio-memristor follows Ohm’s law. Conversely, in the high-resistance state, the smaller conductivity follows the theory of space charge limitation.

**Figure 4:**
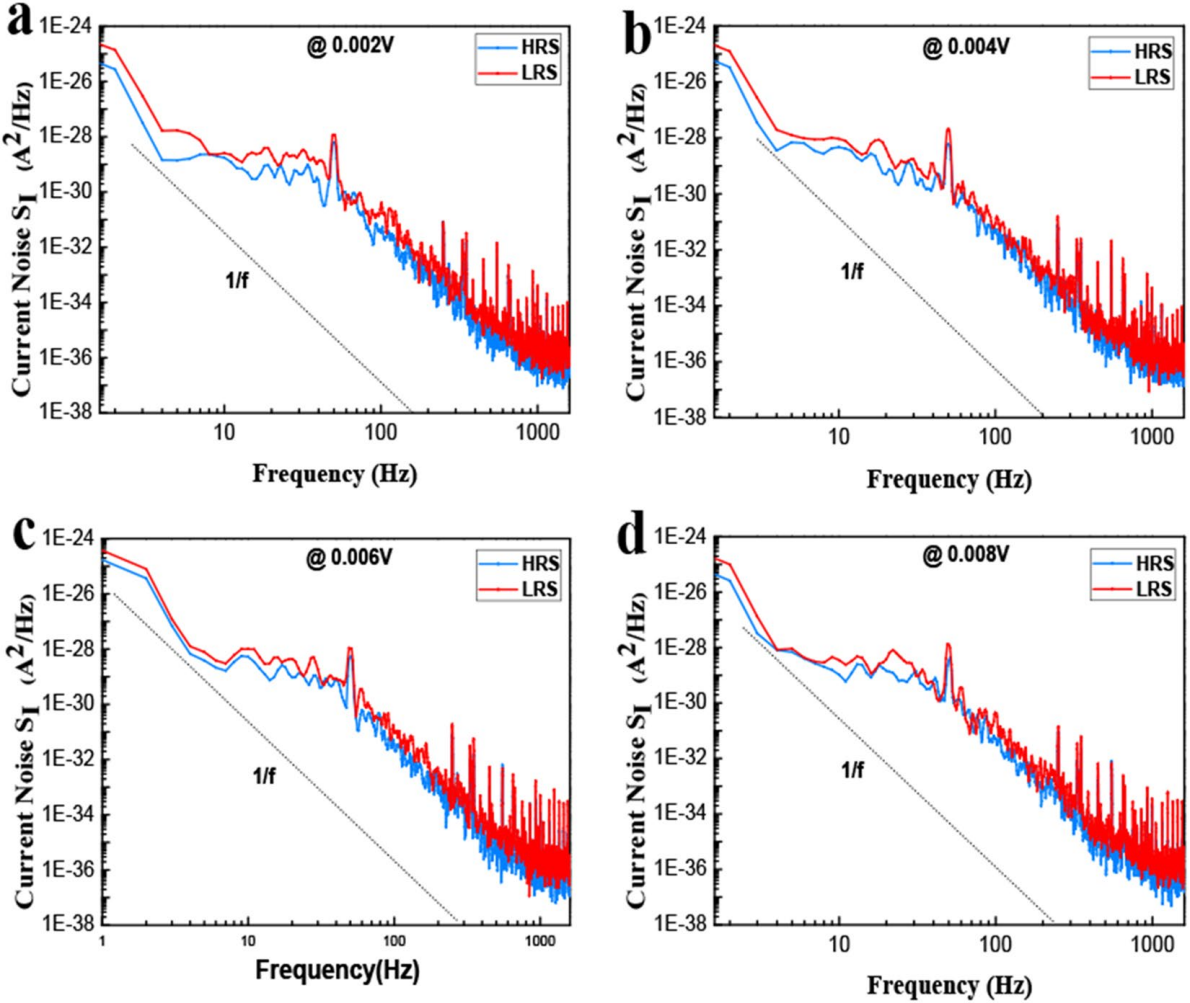
The current noise PSD curves of the silkworm hemolymph memory device in the low- and high-resistance states at various bias voltages.

To understand the resistance switching mechanism of silkworm hemolymph-based bio-memristors, the low-frequency noise of the devices was tested. Figure 4a–d shows the current noise power spectral density (PSD) of the silkworm hemolymph bio-memristor in both low- and high-resistance states at various bias voltages. As shown in Fig. 4a–d, the slopes of all of the curves are approximately 1 in the low- and high-resistance states, and the current noise PSD increases rapidly as the frequency decreases below 1600 Hz in the noise spectrum. The current noise PSD of the silkworm hemolymph bio-memristor in the high-resistance state is lower than that in the low-resistance state. In addition, the current noise PSD of the device is almost independent of the applied voltage, which confirms the inherent nature of the current conduction in the bio-memristor. The slope of each current noise PSD curve is close to 1, which confirms that the noise of the bio-memristor is 1/f noise (flicker noise). Carrier capture/release phenomena are considered to be the main physical source of 1/f noise^4,34^. From the 1/f noise observed in the noise tests, it can be concluded that electron capture and emission occur in the silkworm hemolymph film. The capture and emission of electrons in conductive filaments cause the current in the ITO/silkworm hemolymph/Al bio-memristor to change.

**Figure 5:**
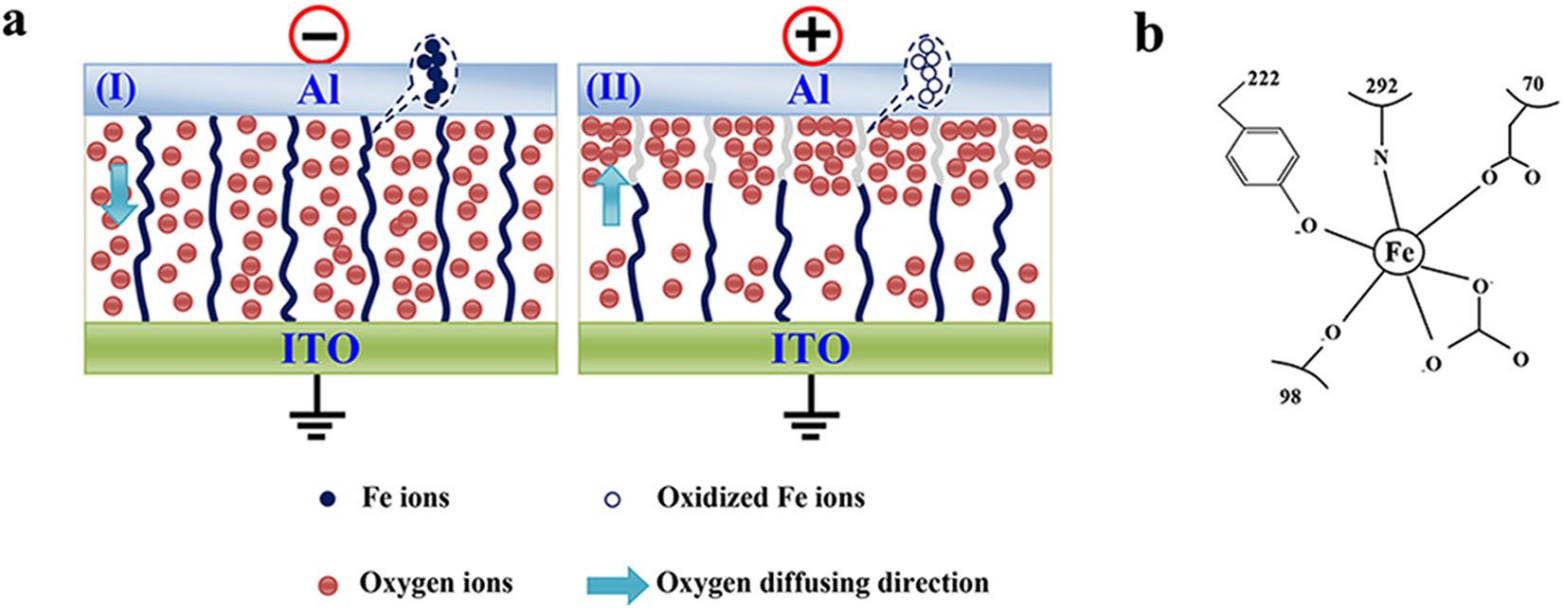
(**a**) Schematic diagram of the resistive switching model for the ITO/silkworm hemolymph/Al bio-memristor. (**b**) The binding of the N-lobes of the silkworm TFs to iron ions.

The migration of oxygen ions and the oxidation and reduction of metal cations in the silkworm hemolymph film under an electric field are the main reasons for the formation and breaking of the conductive filaments. The schematic diagram of the resistive switching model for the ITO/silkworm hemolymph/Al bio-memristor is presented in Fig. 5a. On the one hand, the silkworm has an open circulatory system, and silkworm hemolymph contains proteins, oxygen, carbohydrates, hormones and other substances. The silkworm hemolymph transfers these substances to the organs of the body to meet the needs of silkworm growth and metamorphosis. According to our infrared spectral analysis (Fig. 2a), the oxygen functional groups that are present in the silkworm hemolymph sample include carboxyl groups, carboxyl groups and hydroxyl groups. As shown in Fig. 5a, under a negative voltage, the negatively charged oxygen ions in the silkworm hemolymph film gradually accumulate on the surface of the film near the top electrode, and are more diffuse along the bottom electrode. Conductive filaments are formed, and the device is in a low-resistance state. In contrast, under a positive voltage, the accumulated charges on the top and bottom surfaces are gradually drained, the conductive filaments are broken, and the memory device is in a high-resistance state. Therefore, we conclude that the formation and breaking of the conductive filaments in the ITO/silkworm hemolymph/Al bio-memristor results from the reversible diffusion of oxygen ions in the setting and resetting process. On the other hand, because silkworm hemolymph contains not only iron, calcium, copper and other minerals^35^ but also transferrins (TFs), oxidation-reduction reactions of iron ions occur in this material. Moreover, iron ions can participate in the transfer of electrons between the ITO and Al electrodes because of the relatively small difference between the work functions of iron and the two electrodes. TFs can bind to iron ions, which are widespread in vertebrates and invertebrates. A TF reacts with an Fe^3+^ ion and a bicarbonate ion, and three protons are released. The reaction is as follows^36^:

5 $$F{e}^{3+}+{H}_{6}Tf+HC{{O}_{3}}^{-}\leftrightharpoons {[Fe-{H}_{3}Tf-HC{O}_{3}]}^{-}+3{H}^{+}$$

6 $$F{e}^{3+}+{[Fe-{H}_{3}Tf-HC{O}_{3}]}^{-}+HC{{O}_{3}}^{-}\rightleftharpoons {[F{e}_{2}-Tf-{(HC{O}_{3})}_{2}]}^{2-}+3{H}^{+}$$

Tyrosine (Tyr), glutamine (Gln), threonine (Thr) and aspartic acid (Asp) are usually associated with iron ions. An iron ion binds to Tyr-222, Gln-292, Thr-98, Asp-70 and oxygen atoms from the bidentate carbonate ion in the N-lobes of silkworm TFs^37^. The N-lobes of the silkworm TFs are bound to iron ions, as shown in Fig. 5b. As shown in Fig. 5a, under a negative voltage, the iron ions move to the cathode and obtain electrons. Subsequently, the iron ions are reduced to iron atoms. When the moving iron ions form conductive filaments, the silkworm hemolymph memory device is in a low-resistance state. When the external electric field reverses, the oxidation reaction occurs, and the conductive filaments are broken; as a result, the silkworm hemolymph memory device enters a high-resistance state. The current after conduction should exhibit ohmic characteristics, which is consistent with the test results presented in Fig. 3e. Therefore, in the ITO/silkworm hemolymph/Al bio-memristor, the migration of oxygen ions and the oxidation and reduction of metal cations lead to the formation and breaking of conductive filaments, and this is the resistance switching mechanism of the proposed bio-memristor.

## Conclusion

In summary, we have successfully developed a silkworm hemolymph bio-memristor. To the best of our knowledge, our work is the first to achieve such a device. Experiments revealed that the silkworm hemolymph bio-memristor is a nonvolatile rewritable flash memory device with a current switching ratio exceeding 10^3^. This device can be operated for more than 10^4^ s and remains stable over at least 500 cycles. Measurements of the 1/f noise revealed that the resistance switching characteristics of the silkworm hemolymph bio-memristor are due to the formation and breakage of conductive filaments, which are caused by the migration of oxygen ions and the oxidation and reduction of metal cations in the silkworm hemolymph film. The advantage of silkworm hemolymph bio-memristors is that silkworm hemolymph can be obtained directly from silkworm larvae without the need for complex processes such as purification or synthesis. The silkworm-hemolymph-based nonvolatile resistive memory device described here provides a new pathway for the development and application of bio-memristors.

## Methods

### Device Fabrication

The silkworm hemolymph used in this work was obtained directly from silkworm larvae (*A. pernyi*). The silkworm larvae were obtained from the sericulture base in Northeast China. The ITO glass was washed with deionized water, acetone and isopropanol in sequence (10 min each) in an ultrasonic cleaner. The silkworm hemolymph was spin-coated onto the ITO glass at 3000 rpm for 60 s, and the glass was then dried in a drying oven at 100 °C for 20 min. An aluminum electrode with a thickness of 180 nm was then deposited on the silkworm hemolymph film.

### Characterization

The ITO glass coated with the silkworm hemolymph was imaged using an SEM (Hitachi S3400) and a transmission electron microscope (JEM-2100). The infrared, UV-vis absorption and fluorescence emission spectra of the silkworm hemolymph film were analyzed. Electrochemical analysis was performed on an electrochemical workstation (BAS-100B). The current-voltage characteristics of the silkworm hemolymph bio-memristor were measured using a semiconductor characterization system (Keithley 4200). The low-frequency noise of the devices was tested using a dynamic signal analyzer (Agilent 35670 A).

### Data Availability

All data generated or analyzed during this study are included in this published article.
